# Myocarditis in the Modern Era: Navigating Diagnosis, Innovative Treatments, the COVID‐19 Challenge and the Role of Artificial Intelligence

**DOI:** 10.1155/crp/5956965

**Published:** 2026-08-10

**Authors:** Kevin Mohee, Ibrahim Antoun, Robert Ambrogetti, Mahmoud Eldesouky, Riyaz Somani, André Ng, Sanjay S. Bhandari

**Affiliations:** ^1^ Department of Cardiology, Glenfield Hospital, University Hospitals of Leicester NHS Trust, Leicester, UK, nhs.uk; ^2^ Department of Cardiovascular Sciences, Clinical Science Wing, Glenfield Hospital, University of Leicester, Leicester, UK, le.ac.uk; ^3^ National Institute for Health Research Leicester Research Biomedical Centre, Leicester, UK

**Keywords:** artificial intelligence, cardiac failure, cardiac magnetic resonance, COVID-19, epidemiology, myocarditis

## Abstract

Myocarditis is an inflammatory disease of the myocardium with diverse aetiologies, ranging from infections and autoimmune processes to drug‐induced reactions. Clinical manifestations vary widely from mild chest discomfort to life‐threatening complications such as acute heart failure, cardiogenic shock and sudden cardiac death. Although the diagnosis of myocarditis historically relied heavily on invasive biopsy, recent advances in noninvasive imaging modalities, notably cardiac magnetic resonance (CMR) imaging, have transformed clinical practice. The Lake Louise Criteria (LLC), supplemented by parametric imaging techniques, have significantly enhanced diagnostic accuracy. Furthermore, the emergence of COVID‐19 has renewed interest in myocarditis due to its association with viral‐induced myocardial injury. This review synthesises the current understanding of myocarditis, including epidemiological insights, its diverse aetiologies, diagnostic innovations including the importance of viral load quantification and contemporary management strategies.

## 1. Introduction

According to histopathology, myocarditis is characterised by myocardial inflammation and cardiac myocyte damage. Myocarditis has several possible aetiologies, with common viral infections being the most frequent [1]. During the acute phase of myocarditis, inflammation and oedema can lead to significant left ventricular (LV) dysfunction with acute heart failure (HF), arrhythmias or even sudden cardiac death (SCD) [2]. The clinical spectrum of myocarditis is broad, ranging from mild, transient symptoms to severe and sometimes fatal complications. Recent developments in noninvasive imaging, particularly cardiac magnetic resonance (CMR), have significantly improved diagnostic accuracy, reducing reliance on invasive endomyocardial biopsy (EMB) in routine clinical practice. The COVID‐19 pandemic has further highlighted the importance of understanding myocarditis, as cardiac inflammation has emerged as a critical complication of the SARS‐CoV‐2 infection and the vaccines developed to protect against it. This review provides a comprehensive update on myocarditis, emphasising recent epidemiological trends, improved diagnostic modalities, evolving management strategies and the specific impact of COVID‐19.

### 1.1. Epidemiology

The exact incidence of myocarditis remains difficult to define, as estimates vary substantially according to case definition, diagnostic modality, population studied and ascertainment method. Contemporary population‐level and administrative datasets generally report annual incidence rates in the range of approximately 4–20 cases per 100,000 population, while higher rates are mainly reported in selected autopsy, hospitalised or high‐risk cohorts [3–5]. Histological evidence of myocarditis was found in 22% of patients under 30 years of age and 11% of the remaining patients in an autopsy cohort of patients under 40 years of age who experienced sudden death without known cardiac illness [6]. In a series of autopsies performed on military recruits, myocarditis was found to be responsible for up to 20% of all occurrences of sudden death resulting from undetectable structural cardiac abnormalities [7]. Myocarditis, which can occur in up to 70% of HIV patients, is the most frequent cardiac pathological sign at autopsy [8]. Most studies of acute myocarditis indicate that it is more prevalent in male patients, potentially due to the protective effect conferred by oestrogens on the T‐lymphocyte response. In paediatric populations, myocarditis presents with a higher incidence of acute HF and fulminant progression compared to adults. Enteroviruses, particularly Coxsackie B, remain the predominant viral cause among children [9, 10].

### 1.2. Aetiology

Known aetiologies of myocarditis include a wide spectrum of infectious (Table 1), noninfectious and toxic causes (Table 2), but often the culprit cannot be identified in individual cases [2]. Viruses such as Coxsackie B or common cold viruses are the most common pathogens in western industrialised countries. Still, in developing countries, myocarditis due to bacterial, protozoal or fungal infections is also common. The most frequent cause globally is Chagas disease, a condition caused by infection with the protozoan *Trypanosoma cruzi*, native to Central and South America. In children, enteroviruses such as Coxsackie virus and echovirus are particularly prevalent.

**TABLE 1:** List of infectious causes of myocarditis.

Viral	Adenovirus, Coxsackie A/B virus, cytomegalovirus, dengue, echovirus, Epstein–Barr virus, hepatitis B/C virus, herpes virus, HIV, influenza A and B, mumps, parvovirus, poliovirus, rabies, vaccinia (smallpox vaccine), varicella zoster, yellow fever
Bacterial	*Borrelia* spp., *Chlamydia*, cholera, *Clostridium*, *Corynebacterium*, diphtheria, gonococcal, *Haemophilus*, *Legionella* spp., leptospirosis, meningococcal, *Rickettsia* spp., *Salmonella* spp, *Treponema pallidum*, pneumococcal, *Chlamydia psittaci*, *Mycobacterium tuberculosis*
Protozoal	Amoebiasis, *Trypanosoma cruzi*, leishmaniasis, malaria, toxoplasmosis

**TABLE 2:** List of noninfectious and toxic causes of myocarditis.

Cardiotoxins	Alcohol, anthracyclines, arsenic, catecholamines, carbon monoxide, cocaine, cyclophosphamide, chemotherapy drugs, lithium, irradiation, hypothermia, heavy metals (copper, lead, iron)
Hypersensitivity/allergic reactions	Antibiotics (penicillin, cephalosporin, sulphonamide), clozapine, diuretics (thiazide, loop), dobutamine, insect bites, antirheumatic drugs
Systemic disorders	Coeliac disease, SLE, Wegener’s granulomatosis, Churg–Strauss syndrome, Takayasu arteritis, giant cell myocarditis, sarcoidosis, scleroderma, inflammatory bowel disorders, Kawasaki disease, thyrotoxicosis

Low‐level detection of viral genomes (e.g., parvovirus B19 < 500 copies/μg DNA) in EMB specimens often reflects latent infection rather than active pathogenic involvement. Furthermore, nV‐6, a chromosomally integrated form of human herpesvirus 6, can be detected at high copy numbers without pathogenicity, necessitating cautious interpretation of PCR results [11].

Myocardial inflammation may also occur as part of a systemic illness or with granulomatous and autoimmune conditions such as Churg–Strauss, sarcoidosis, giant cell myocarditis (GCM), systemic lupus erythematosus (SLE) and thyrotoxicosis. However, these are less common aetiologies than viral myocarditis or postinfection inflammatory cardiomyopathy [12]. Finally, medications or toxins can cause myocarditis directly by affecting the heart tissue or by triggering hypersensitivity reactions, which are frequently connected to eosinophilic myocarditis. Eosinophilic myocarditis can also be associated with hypereosinophilic syndromes such as Churg–Strauss or caused by helminthic and parasitic infections.

### 1.3. Pathogenesis

Few human tissue studies of myocarditis exist; therefore, most evidence on the molecular pathogenesis of viral and autoimmune myocarditis comes from rodent models and isolated cell systems [8]. The exact mechanism and pathway of heart infection are unknown for many viruses. Acute myocardial infection in these mouse models develops into myocardial damage upon exposure to intracellular antigens, such as cardiac myosin, which trigger the innate immune response. Weeks later, specialised immunity mediated by T cells and antibodies that target infections and other endogenous cardiac epitopes leads to significant inflammation. Most patients experience a brief immunological response with minor aftereffects, and the infections eventually resolve. On the other hand, the virus continues to infect other patients, resulting in long‐term myocyte destruction and myocarditis [8]. In addition to direct viral cytotoxicity, autoimmunity and dysregulated inflammatory cascades contribute significantly to myocardial injury in chronic phases. Genetic predisposition may play a role in susceptibility to immune‐mediated injury. This evolving understanding has prompted new interest in immunomodulatory therapies, contingent upon exclusion of active viral infection [13–15].

Eosinophilic myocarditis can be linked to endomyocardial fibrosis, hypersensitivity myocarditis (HSM), parasitic infection and idiopathic hypereosinophilic syndrome. Common features of these diseases include mature eosinophil infiltration in multiple organs, including the heart, and peripheral eosinophilia.

An immunological reaction in the heart that is frequently drug‐related is called HSM, which is a kind of eosinophilic myocarditis. HSM is typically temporally associated with a medicine that has just been started. Many medications, including ampicillin, methyldopa, furosemide, hydrochlorothiazide, tetracycline, azithromycin, aminophylline, phenytoin, benzodiazepines and tricyclic antidepressants, have been linked to the HSM [12, 16]. HSM does not, however, always appear at an early stage of drug usage. It has been documented that myocarditis occurs in patients receiving the antipsychotic medication clozapine more than 2 years after the medication was started [17]. In an autopsy study, 16 cases (< 0.5%) were identified in > 3000 consecutive autopsies [18].

## 2. Classification of Myocarditis

A clinical‐pathologic classification using a combination of histologic and clinical characteristics may prognosticate patients with HF resulting from myocarditis. Lieberman et al.’s classification may be used to differentiate various forms of myocarditis [19].

We propose an updated four‐axis classification system that integrates the following:

1. Clinical presentation (e.g., chest pain syndrome, acute HF, arrhythmias or cardiogenic shock)
2. Histopathology (e.g., lymphocytic, eosinophilic, giant cell or sarcoid granulomatous)
3. Aetiology (e.g., viral, autoimmune, drug‐induced, toxic, post‐COVID‐19 infection or vaccination)
4. Time course (acute, subacute, chronic or relapsing)

Emerging subtypes, including immune checkpoint inhibitor (ICI)–associated myocarditis, warrant specific classification due to their unique pathophysiology, overlapping skeletal muscle involvement (e.g., myositis or myasthenia‐like syndromes) and need for prompt corticosteroid therapy. Fulminant myocarditis (FM), characterised by rapid progression to cardiogenic shock, should also be recognised as a high‐risk clinical category demanding early mechanical circulatory support consideration.

### 2.1. Acute Myocarditis

A total of 75% of patients with acute chest pain, elevated serum troponin levels at presentation and no‐flow‐limiting coronary arteries at invasive catheterisation are diagnosed with acute myocarditis [20]. Acute myocarditis may also cause LV systolic dysfunction and progress to dilated cardiomyopathy (DCM).

### 2.2. Chronic Active Myocarditis

Chronic active myocarditis is characterised as presenting with a less distinct onset of symptoms but associated with frequent clinical and histologic relapses, progression to HF with chronic inflammatory changes and mild to moderate fibrosis on EMB.

### 2.3. Chronic Persistent Myocarditis

Chronic persistent myocarditis is characterised as presenting with a less distinct onset of symptoms with a persistent infiltrate, often with foci of myocyte necrosis in the absence of HF despite continuous chest pain or palpitation.

### 2.4. FM

FM is characterised by sudden onset HF, hypotension and the rapid development of cardiogenic shock, often requiring inotropes and mechanical circulatory support (e.g., venoarterial extracorporeal membrane oxygenation [VA‐ECMO]) [10, 21]. It can mimic acute coronary syndrome with ST elevation and troponin rise, but coronary angiography typically reveals normal arteries [22]. Prognosis in FM can be favourable with early diagnosis and aggressive supportive care [21].

ICI‐associated myocarditis typically presents within weeks of ICI initiation and may manifest with fatigue, dyspnoea, chest pain or asymptomatic ECG/troponin abnormalities. It has a high morbidity and mortality risk, particularly when coexisting with myositis or myasthenia‐like symptoms [21, 23]. The incidence may be as high as 1%, with a mortality rate approaching 50% in severe cases. Early initiation of corticosteroids is essential, and immunosuppressive agents (e.g., mycophenolate and infliximab) may be considered for steroid‐refractory disease [21].

### 2.5. GCM

It is distinguished by high‐degree heart block, dilated LV, new ventricular arrhythmia, ventricular systolic dysfunction and/or inability to respond to standard HF treatment in a time frame of 1–2 weeks. Of all the forms, its prognosis is the worst [24].

## 3. Clinical Presentation

Myocarditis can mimic myocardial infarction, presenting with acute chest pain, arrhythmia or rapidly progressive HF. Many patients initially have nonspecific symptoms; therefore, cardiac involvement may not be recognised early. Typically, cardiac involvement is only considered when symptoms such as exertional dyspnoea, palpitations and chest pain start to manifest [2]. Often, the patients describe chest pain that is worse on inspiration, better when leaning forward (pericarditis), instead of angina.

The European Study of Epidemiology and Treatment of Inflammatory Heart Disease screened 3055 patients with suspected acute or chronic myocarditis, with 72% having dyspnoea, 32% having angina and 18% having arrhythmias [25]. The clinical presentation of myocarditis differs from that in adults; children have a more sudden and severe presentation [26].

HSM is usually characterised by acute rash, fever and peripheral eosinophilia; however, some patients present with sudden death or rapidly progressive HF.

Necrotising eosinophilic myocarditis has an exceptionally poor prognosis, with most cases diagnosed at autopsy. In contrast, eosinophilic myocarditis associated with the hypereosinophilic syndrome typically evolves over weeks to months. The presentation is usually biventricular failure, although arrhythmias may lead to sudden death.

## 4. Diagnostic Modalities

The clinical heterogeneity of myocarditis makes diagnosis challenging. There is no gold standard diagnostic test with perfect sensitivity and specificity [8, 27, 28]. Diagnosis is typically made using a combination of clinical features, laboratory findings, cardiac imaging and histological analysis when required [22, 27].

### 4.1. Physical Examination

Findings on clinical examination are variable and can mimic other noninflammatory cardiac disorders but may shed light on the underlying cause. These can include sustained ventricular tachycardia or new heart block with rapidly progressive congestive HF–GCM, displaced apex beat (ventricular dilation), soft S1 (mitral regurgitation), S3 or S4 gallop (HF), lymphadenopathy (sarcoidosis), polyarthritis, subcutaneous nodules or erythema marginatum (acute rheumatic fever) [29].

### 4.2. Electrocardiogram (ECG)

The commonest ECG abnormality is sinus tachycardia with nonspecific ST–T wave changes [30]. Supraventricular and ventricular arrhythmias can also be observed, as can *Q* waves or bundle branch block, which is associated with increased rates of heart transplant (HT) or mortality. Occasionally, a pseudo‐infarct and ischaemic changes are present. Pericarditis can often be clinically associated with myocarditis but manifests as pericarditis‐like changes on the ECG. The ECG sensitivity is 47% in myocarditis [31].

### 4.3. Biomarkers

Cardiac‐specific serum biomarkers such as troponin I or *T* can help diagnose myocarditis and can also be used to monitor clinical progression. In patients with clinically suspected myocarditis, a troponin *T* cutoff > 0.1 has shown a sensitivity, specificity and positive predictive value of 53%, 94% and 93%, respectively, following a normal angiogram [32]. Smith et al. also examined the value of troponin I in a subgroup of the Multicenter Myocarditis Treatment Trial. They found a low sensitivity (34%) of elevated troponin I for the entire group but a high specificity (89%). Also, a short duration of symptoms (< 4 weeks) was associated with a significantly higher sensitivity for detecting biopsy‐proven disease [33]. Most clinicians now routinely measure either troponin *T* or *I* whenever a clinical diagnosis of myocarditis is considered [34]. Elevation of cardiac biomarkers occurs in 32% of patients with acute pericarditis and probable myopericarditis but increases to 70% in hospitalised patients. Cardiac troponin elevation is transient and slightly above the limit [35, 36]. CK and CK‐MB are less specific and have low predictive value.

### 4.4. Echocardiography

Echocardiography is an important tool for diagnosing and prognosticating myocarditis by assessing LV function and excluding other causes of HF [25]. Classical hallmarks of myocarditis on echocardiography include global hypokinesis with or without pericardial effusion, and in some cases, regional wall motion abnormalities can mimic myocardial infarction. Increased sphericity and LV volume were observed in chronic active myocarditis in the Myocarditis Treatment Trial [37]. Felker et al. [36] developed echocardiographic criteria to facilitate differentiation between FM and acute myocarditis. Patients with acute myocarditis have a less severe degree of myocardial inflammation, less severe haemodynamic compromise and an indistinct onset of symptoms. FM is characterised by a distinct viral prodrome, the sudden onset of severe haemodynamic compromise and marked myocardial inflammation. Patients who presented with acute myocardial oedema, near‐normal LV diastolic dimensions but increased septal thickness were those with FM, while those who presented with acute myocarditis had increased diastolic dimensions but normal septal thickness [38]. When comparing patients with acute myocarditis to those with FM, ventricular function significantly improved after 6 months [35]. Furthermore, Caspar et al. [39] have shown that with the advent of novel imaging modalities such as 2D and three‐dimensional (3D) speckle‐tracking (ST) echocardiography, myocardial dysfunction after an acute episode of myocarditis can be detected despite a preserved left ventricular ejection fraction (LVEF), thus adding incremental information over conventional TTE.

### 4.5. CMR

Myocarditis was formerly diagnosed by exclusion or invasive biopsy; however, CMR imaging, which includes gadolinium enhancement and T2‐weighted imaging, has demonstrated good diagnostic accuracy. The Lake Louise Criteria, a consensus set of standards for CMR assessment of myocarditis, were established in 2009 [40]. Based on two of the three ‘Lake Louise’ criteria, myocarditis can be diagnosed with CMR: (a) increased T2 ratio ≥ 2.0 on T2‐weighted images, or global or regional myocardial signal intensity increase; (b) increased global myocardial early gadolinium enhancement (EGE) ratio ≥ 4.0 on EGD; and (c) at least one focal, nonischaemic lesion at inversion‐recovery late gadolinium enhancement (LGE) MR imaging [40]. Native T1 mapping may be more sensitive than LGE and conventional T2‐weighted imaging for detecting acute myocarditis. In contrast to LGE imaging, native T1‐mapping can show the characteristic nonischaemic damage patterns in acute myocarditis without using contrast agents [41].

In the acute phase, T2W imaging can identify regions of myocardial oedema as high signal intensity, typically in the subepicardium or mid‐wall. More recently, T1, ECV and T2 mapping techniques have superseded conventional T2‐weighted imaging for detecting myocardial oedema and inflammation and thus may be used together with LLC [41]. Native T1, T2 and extracellular volume (ECV) fraction mapping are newer CMR parametric imaging techniques that identify tissue changes associated with myocarditis without using an exogenous contrast agent. Greater T1 and T2 increases may be seen with acute rather than subacute inflammation [41]. In the MyoRacer Trial, which involved biventricular myocardial biopsies, LLC demonstrated inferior diagnostic performance to native T1, ECV and T2 mapping [42]. A systematic review and meta‐analysis comparing the diagnostic accuracy of CMR index tests for acute myocarditis included 22 studies. They revealed that only native T1 offered a significantly better sensitivity than LLC, and native T1, T2 and ECV mapping provide comparable diagnostic performance to LLC [43].

In acute myocarditis, rupture of myocyte membranes permits gadolinium to diffuse into the cells, resulting in an increased tissue‐level concentration and subsequent contrast enhancement on EGE imaging. Ideally, patients with acute myocarditis should be scanned within 2 weeks as the sensitivity of CMR drops substantially after this period. Necrosis and fibrosis resulting from irreversible tissue injury are demonstrated by LGE. Unlike coronary disease, which usually affects the subendocardial region, although the scar can be full thickness, the characteristic pattern on LGE imaging is hyperenhancement in the subepicardium with variable progression toward the mid‐wall and typical sparing of the subendocardium [44, 45]. Although the usual hyperenhancement pattern is a significant signal of myocarditis, the diagnosis is not completely ruled out without the pattern. The most often affected walls are the lateral and inferolateral ones. However, myocarditis caused by human herpesvirus 6 or parvovirus B19 has been linked to a distinct pattern of septal mid‐wall hyperenhancement [40].

In practice, CMR imaging is the first‐choice noninvasive imaging test after echocardiography in the vast majority of patients presenting with myocarditis (compatible symptoms with raised troponin, LV systolic dysfunction on echocardiography and normal coronary arteries).

Thus, CMR plays a key role in distinguishing myocarditis from myocardial infarction, which has a different contrast enhancement pattern on LGE imaging and always involves the subendocardium. In an acute inpatient setting of acute coronary syndrome without obstructive coronary disease, the most important contribution of CMR is the discrimination of myocarditis from myocardial infarction or takotsubo cardiomyopathy. MRI may also guide tissue sampling during EMB [45].

**FIGURE 1:**
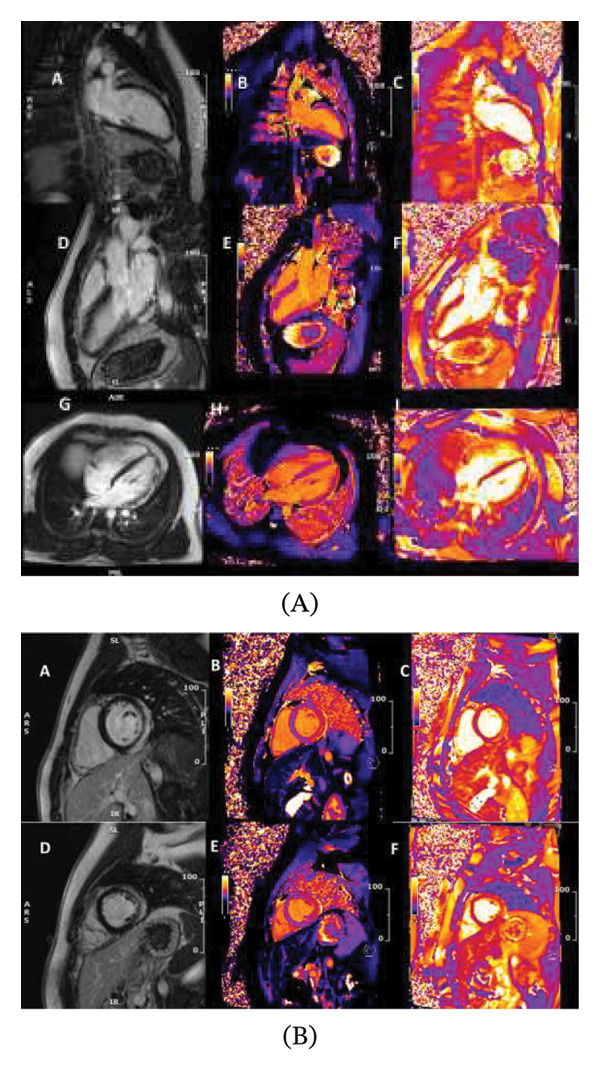
Panel A: (A) 2ch LGE, (B) 2ch T1, (C) 2ch T2, (D) 3ch LGE, (E) 3ch T1, (F) 3ch T2, (G) 4ch LGE, (H) 4ch T1 and (I) 4ch T2. Panel B: (A) SAX basal LGE, (B) SAX basal T1, (C) SAX basal T2, (D) SAX mid LGE, (E) SAX mid T1 and (F) SAX mid T2.

Lastly, CMR may also aid risk stratification in viral myocarditis. In a study of 203 consecutive patients with biopsy‐proven myocarditis, regardless of LVEF, there were no incidences of SCD in patients without hyperenhancement on LGE imaging [46]. Therefore, the absence of hyperenhancement may help identify patients at reduced risk who can be conservatively followed up on without considering using an implantable cardioverter‐defibrillator (ICD). To validate these results, large prospective investigations are necessary. Representative CMR sequences demonstrating typical myocarditis findings are shown in Figure 1A‐B.

### 4.6. Myocardial Scintigraphy

Gated myocardial perfusion scintigraphy (MPS) can assess cardiac function and perfusion and has been used in myocarditis diagnosis [47]. Two studies have reported a good correlation between the degree of perfusion as a marker of myocarditis and ST–T changes and myocardial enzyme levels. Lee et al. and de Winter et al. [48, 49] reported similar findings, showing that gated MPS provides further data and clinical and perfusion information for predicting cardiac death.

### 4.7. EMB

Histological evidence is still required to definitively confirm a diagnosis of myocarditis. Hence, EMB remains the diagnostic gold standard, providing immunohistological (IMH) confirmation and molecular analysis that cannot be achieved with noninvasive testing [50]. This aids in precise aetiological diagnosis, distinguishing immune‐mediated and infectious causes and guiding tailored antiviral and immunosuppressive treatments [51].

**FIGURE 2:**
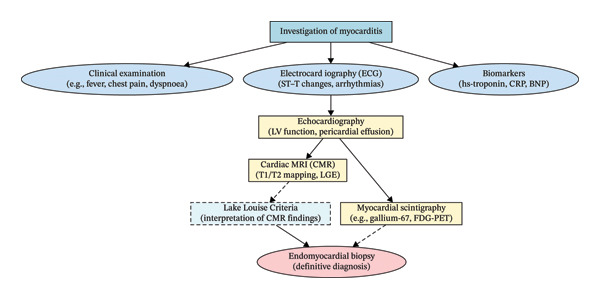
Flowchart summarising investigations for suspected myocarditis. BNP, B‐type natriuretic peptide; CMR, cardiac magnetic resonance; CRP, C‐reactive protein; ECG, electrocardiography; FDG‐PET, fluorodeoxyglucose positron emission tomography; hs‐troponin, high‐sensitivity troponin; LGE, late gadolinium enhancement; LLC, Lake Louise Criteria; LV, left ventricle.

EMB is not routinely performed in all suspected cases of myocarditis. Given its invasive nature, EMB is limited to carefully selected fulminant or treatment refractory presentations in which the anticipated diagnostic, prognostic or therapeutic yield is judged to outweigh the procedural risks [50]. Its use in real‐world practice is reported to range from 0% to 44% of adult cases with high‐risk or FM [52–54]. In most nonfulminant cases, once coronary artery disease and other potential differentials are excluded, noninvasive evaluation with CMR is often the initial diagnostic investigation [50]. A pragmatic diagnostic pathway reflecting this approach is summarised in Figure 2.

The American Heart Association (AHA)/American College of Cardiology Foundation (ACCF)/European Society of Cardiology (ESC) joint scientific statement recommended that EMB should be carried out in patients with HF and (i) a normal‐sized or dilated LV, < 2 weeks of symptoms and haemodynamic instability for suspected FM and also in (ii) patients with a dilated ventricle, 2 weeks–3 months of symptoms, new ventricular arrhythmias or Mobitz Type II second‐degree or complete heart block not responding to usual treatment within 1–2 weeks [51].

The FULLMOON study was an international, multicentre retrospective cohort study of over 400 adults with suspected FM admitted to intensive care (ITU) across 15 different countries [53]. It found that early EMB (defined as within 2 days of ITU admission) was independently associated with a lower rate of death, HT or LVAD at 1 year (odds ratio 0.44; 95% CI 0.22–0.86; *p* = 0.016). Survival free of HT or LVAD at 1 year was seen in 63% of patients with early EMB as compared to 40% to those with EMB after 2 days of ITU admission (odds ratio 0.44, 95% CI 0.22–0.86; *p* = 0.021). These findings are consistent with the potential clinical value of early EMB in appropriately selected fulminant presentations, while recognising that FULLMOON was a retrospective observational study and should be interpreted in that context. Nevertheless, they support recent guidelines and the concept that early EMB in cases of FM can facilitate timely escalation of treatment with steroids and other aetiology‐specific immunosuppression when indicated [53]. The FULLMOON study also proposed a practical diagnostic algorithm combining early EBM, advanced imaging with CMR and positron emission tomography (PET) and multiplex PCR diagnostics. This approach improves diagnostic sensitivity and specificity, especially in complex cases such as ICI myocarditis or FM [55]. Broad implementation of this algorithm may help reduce misdiagnosis and facilitate earlier targeted treatment.

EMB use should be guided by multidisciplinary teams and performed in centres with appropriate expertise [50]. Given the nonuniform nature of inflammation in myocarditis, several key practical considerations can reduce sampling error in EMB. It is recommended to take 5–10 biopsy samples, depending on the distribution of inflammation [56]. The combination of CMR and electroanatomic mapping to identify sampling sites has been suggested to increase EMB sensitivity [56]. Early EMB, defined as within 2 weeks of symptom onset, can increase the likelihood of detecting active inflammation and reduce the risk of false‐negative results [57]. The addition of IMH and PCR to conventional histology enables highly sensitive aetiologic characterisation, aiding personalised therapy [50].

### 4.8. Role of MicroRNAs (miRNAs)

miRNAs are small, single‐stranded RNA sequences of approximately 20–24 nucleotides in length that regulate gene expression on a posttranscriptional level. The role of miRNAs in human myocarditis is not fully understood. However, two identified miRNAs, miR‐208b and miR‐499, are elevated and can be detected in plasma in viral myocarditis patients. As a result, they might be utilised to diagnose myocarditis and to assess severity. All patients have elevated levels of miR‐499, but only fulminant virus–induced myocarditis was shown to express both miR‐208b and miR‐499 [58].

## 5. Treatment of Myocarditis

**FIGURE 3:**
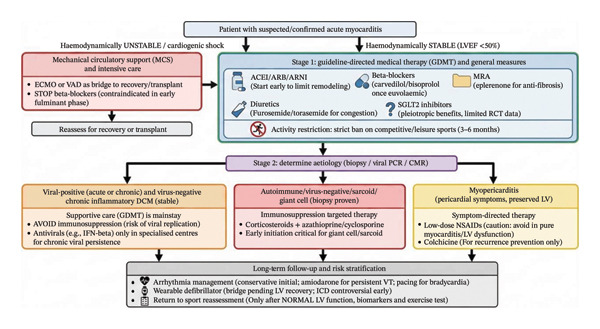
Concise clinical framework for myocarditis management. ECMO = extracorporeal membrane oxygenation, VAD = ventricular assist device, ACEi = angiotensin‐converting enzyme inhibitors, ARB = angiotensin II receptor blockers, ARNI = angiotensin receptor–neprilysin inhibitor, MRA = mineralocorticoid receptor antagonist, SGLT2 = sodium–glucose cotransporter 2, RCT = randomised controlled trial, PCR = polymerase chain reaction, CMR = cardiovascular magnetic resonance, DCM = dilated cardiomyopathy, IFN‐beta = interferon beta, LV = left ventricle, NSAIDs = nonsteroidal anti‐inflammatory drugs, VT = ventricular tachycardia, ICD = implantable cardioverter defibrillator.

According to evidence‐based guidelines, the mainstay of management, regardless of aetiology, is the treatment of underlying HF and/or arrhythmia. Given the limited number of myocarditis‐specific randomised trials, therapeutic recommendations frequently rely on observational myocarditis cohorts, small subtype‐specific studies, preclinical data and extrapolation from broader HF evidence; therefore, reported effect estimates should be interpreted in the context of study design, population size and applicability to myocarditis [59–61]. A practical staged management framework is summarised in Figure 3. The specific treatment depends on the results of a myocardial biopsy. The precise causes of the disease and the degree of irreversible tissue damage at the beginning of therapy determine how well both acute and chronic myocarditis respond to treatment.

### 5.1. Nonsteroidal Anti‐Inflammatory Drugs and Colchicine

In contrast to pericarditis, for which aspirin or NSAIDs are the treatment of choice, NSAID therapy for myocarditis is ineffective. Experimental data suggest that NSAIDs may worsen myocardial injury [62]. Thus, low‐dose NSAID therapy is only recommended in myocarditis if there is significant pericardial involvement (myopericarditis) with related symptoms and provided that there is no significant LV dysfunction. Treatment with colchicine is valuable in cases of recurrence of acute pericarditis, as this is the only drug that has been shown to reduce the recurrence rate. However, there is no specific role for colchicine in myocarditis in the absence of pericardial involvement. Colchicine may protect the myocardium in Chagas disease, as indicated by decreased interstitial myocardial fibrosis [63]. Corticosteroids should be avoided unless there is a specific autoimmune aetiology, as they are independently associated with recurrence [64].

Studies suggest that mechanical circulatory support, such as ventricular assist devices or extracorporeal membrane oxygenation, can be used as a bridge to heart transplantation or recovery in patients whose condition worsens despite optimal medical therapy [65].

### 5.2. Antiviral Therapy

Data from antiviral treatment studies are limited and mostly restricted to animal models.

#### 5.2.1. Early‐Stage Antiviral Therapy

Antiviral therapy with ribavirin or interferon‐alpha reduces the severity of myocardial lesions and mortality in experimental murine myocarditis caused by the Coxsackie B3 virus. However, this beneficial effect is seen only if therapy is started before inoculation or soon thereafter [46, 66]. Therefore, the applicability of these findings to humans is uncertain since patients with viral myocarditis are usually not seen in the earlier stages. These agents, therefore, are of limited use in virus‐associated heart disease.

#### 5.2.2. Chronic Stage Antiviral Therapy

Interferon beta, administered to a cohort of 22 patients with myocardial enteroviral or adenoviral persistence and LV dysfunction, eliminated the viral genomes in all patients and improved LV function in 15 of 22 patients [67].

In the BICC (Betaferon in chronic viral cardiomyopathy) trial, a Phase II study, 143 patients with inflammatory DCM and confirmed myocardial viral infection were treated with interferon beta‐1b (IFN‐beta‐1b) versus placebo. Treatment with IFN‐beta‐1b significantly reduced the viral load in the myocardium; however, complete viral elimination was not achieved in all patients. Various parameters were evaluated, but only the NYHA functional class and patient global assessment improved [68].

### 5.3. Immunosuppressive Treatment

In the Myocarditis Treatment Trial, 111 patients with a histopathologic diagnosis of myocarditis and LVEF < 45% were randomly assigned to receive conventional therapy alone or immunosuppressive treatment, which consisted of prednisone with either cyclosporine or azathioprine for 28 weeks. In all patients, the LVEF improved from 25% to 34%, and the mortality rate was 20% at one year and 56% at 4.3 years. There was no difference in outcome in the two treatment groups [69].

Parrillo et al. examined the efficacy of prednisone in patients with idiopathic DCM [70]. Only patients with congestive HF because of DCM were selected; patients with underlying coronary, hypertensive or congenital heart disease, valvular disease and a history of chronic alcohol excess, were excluded. For this reason, 102 patients were randomly assigned to either treatment with prednisone (60 mg per day) or a control group. The increases observed in the ejection fraction during prednisone treatment were small, their duration was limited, and the side effects were significant. The administration of prednisone was not recommended as standard therapy for DCM [70].

In contrast to acute myocarditis, patients with evidence of chronic inflammation may respond to immunosuppressive therapy. Eighty‐four patients with DCM of greater than 6 months duration and evidence of chronic inflammation on biopsy were randomly assigned to receive either immunosuppression or placebo for 3 months and then were followed for 2 years. There was no difference in the primary endpoint (death, heart transplantation or hospital readmission) at two years (23 versus 21% for placebo). However, patients treated with immunosuppressive therapy had significantly improved LVEF and clinical status [71].

The double‐blinded TIMI (Immunosuppressive Therapy in Patients with Virus‐Negative Inflammatory Cardiomyopathy) trial randomly assigned 85 patients with chronic (> 6 months) stable HF but with no evidence of myocardial viral genomes to receive either azathioprine and prednisone or placebo for 6 months. In 88% of patients on immunosuppressive therapy, LVEF improved by > 10%, while LVEF did not improve in patients in the placebo group. Patients in the immunosuppressive treatment showed better clinical improvement with significantly lower average New York Heart Association classes at 6 months [72].

Granulomatous myocarditis with a fulminant course is usually identified at postmortem. Still, its prognosis can be improved if immunosuppressive treatment is initiated early, especially if the underlying aetiology is autoimmune or sarcoid [73].

**FIGURE 4:**
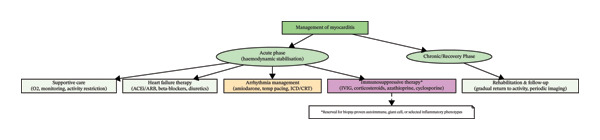
Flowchart summarising management of suspected myocarditis. ACEi, angiotensin‐converting enzyme inhibitor; ARB, angiotensin receptor blocker; CRT, cardiac resynchronisation therapy; ICD, implantable cardioverter‐defibrillator; IVIG, intravenous immunoglobulin.

A pragmatic framework is outlined in Figure 4, while Table 3 provides a concise subtype‐based treatment matrix, including viral‐positive, virus‐negative inflammatory, ICI‐associated, eosinophilic and GCM.

**TABLE 3:** Myocarditis treatment by subtype.

Subtype	First‐line	Escalation/adjunct	Devices & MCS
Acute lymphocytic	If heart failure or LVSD GDMT HF tx NSAIDs: AVOID (mortality risk) (only if myopericarditis + preserved LVEF) Colchicine: only if pericarditis	IS: withhold if viral PCR+ Virus‐negative consider after MDT: AZA + pred (TIMIC; 88% LVEF improvement) GDMT for HF as per EF	After MDT consider: WCD if severe LVSD (bridge), ICD deferral and reassess at 3–6/12 Temp pacing if CHB

Fulminant	Haemodynamic stabilisation in ITU IV inotropes (hold BB until shock resolved) early EMB ≤ 48h (FULLMOON: OR 0.44 death/HT/LVAD) GDMT as tolerated	Aetiology‐guided IS once viral Ax excluded IV methylpred if ICI/GCM/autoimmune	Consider VA‐ECMO for refractory CS Consider Impella if LV overload LVAD or HT if no recovery^∗^

Giant Cell (GCM)	After specialist review and confirmed diagnosis ‐ Methylpred/pred (high‐dose) ± Cyclosporine ± AZA (triple IS) GDMT concurrently as tolerated	Consider with specialist review in specialist centres early HT (treatment of choice) GCM recurs post‐HT (∼26% at 3 yrs) Lifelong IS posttransplant	Consider the following with specialist review Perm pacing for high‐degree AVB ICD for refractory VA VA‐ECMO/LVAD as bridge to HT^∗^

Eosinophilic	Stop causative agent or drug/Treat underlying cause (HES/EGPA/parasites) IV methylpred (500–1000 mg/day for 1–3 days) or oral Pred 1–2 mg/kg/day	Consider aetiology specific IS with specialist review for HES, EGPA ‐ cyclophosphamide, imatinib, mepolizumab; Antiparasitic therapy essential for proven aetiology	Early referral to specialist centre ‐ VA‐ECMO/LVAD for CS^∗^ ICD if sustained VA

ICI‐associated	Hold ICI IV methylpred (500–1000 mg daily for 3 days) or oral pred 1–2 mg/kg/day—maintain dose until clinic/biomarker improvement then taper for 4–6 wks tx possible associated myasthenia, myositis	With specialist review escalate IS: ‐ MMF, Antithymocyte globulin, tacrolimus, ‐ IVIG, plasmaphaeresis Infliximab: CAUTION if LV dysfunction Permanent ICI discontinuation (mod‐severe)	VA‐ECMO/LVAD for refractory CS^∗^ Temp pacing if CHB ICD if sustained VA

COVID‐19/post‐COVID	Standard GDMT Postvaccination (mild): supportive ± NSAIDs/colchicine if myopericarditis	IS (steroids/IVIG) only if fulminant immune‐mediated Colchicine if pericarditis component	VA‐ECMO/MCS for fulminant CS^∗^ Temp pacing if conduction block ICD/WCD: defer; reassess LV

Autoimmune/systemic disease	Treat underlying disease (SLE/EGPA/Kawasaki/thyrotoxicosis) Corticosteroids (aetiology‐guided) Kawasaki: IVIG 2 g/kg x1 dose + aspirin Thyrotoxicosis: carbimazole + BB	HCQ (SLE)^∗^ MTX, AZA, MMF, rituximab, cyclophosphamide (disease‐specific)^∗^ Thyrotoxicosis: often resolves with euthyroid state	ICD/CRT per standard HFrEF criteria Temp pacing if advanced AVB VA‐ECMO/MCS if CS^∗^

*Note:* tx, treatment; NSAIDs, nonsteroidal anti‐inflammatory drugs; IS, immunosupression; MDT, multidisciplinary team; ITU, intensive care unit; IV, intravenous; EMB, endomyocardial biopsy; Ax, aetiology; ICI, immune check point inhibitor; IVIG, intravenous immunoglobulin; HES, hypereosinophilic syndrome; EGPA, eosinophilic granulomatosis with polyangiitis; MMF, mycophenolate mofetil; HCQ, hydroxychloroquine; MTX, methotrexate; AZA, azathioprine; ECMO, extracorporeal membrane oxygenation.

Abbreviations: BB, beta blocker; CHB, complete heart block; GDMT, guideline‐directed medical therapy; HF, heart failure; HT, heart transplant; ICD, implantable cardioverter‐defibrillator; LVAD, left ventricular assist device; LVEF, left ventricular ejection fraction; LVSD, left ventricular systolic dysfunction; SLE, systemic lupus erythematosus; VA, ventricular arrhythmia; WCD, wearable cardioverter, defibrillator.

^∗^Requires specialist centre input or after specialist review.

### 5.4. Treatment for Arrhythmia

Arrhythmias in patients with acute myocarditis are usually treated with supportive care. They typically terminate after the acute phase of the disease, which can last several weeks. AHA/ACCF/ESC guidelines recommend conservative arrhythmia management in myocarditis patients [64]. Outside the acute phase, arrhythmia management should align with current ESC guidelines on arrhythmia and device implantation.

Amiodarone may be required for patients with symptomatic or persistent ventricular arrhythmias. In acute myocarditis, temporary pacing may be necessary for patients with symptomatic bradycardia or complete heart block. The indications for an ICD are controversial, as myocarditis may heal completely, or LV function may improve significantly with guideline‐based HF therapy. In the setting of likely reversible LV dysfunction associated with acute myocarditis, a wearable external defibrillator vest may be used as a bridge awaiting reassessment of LV function [74] However, patients should be assessed on an individual basis.

### 5.5. HF Therapy

Myocarditis with acute DCM should be treated per AHA/ACCF/ESC guidance [59–61]. LV dysfunction should be managed with standard HF therapy: an ACE inhibitor or ARB, beta‐blockade if haemodynamically tolerated and diuretics when clinically indicated.

#### 5.5.1. ACE Inhibitors/ARBs

Early renin–angiotensin blockade may limit progression and remodelling. In mouse autoimmune or viral myocarditis, ACEI (captopril) and ARBs (losartan and olmesartan) reduced inflammation, necrosis and fibrosis [75–77]. In rat autoimmune myocarditis–induced DCM, olmesartan improved LV function and remodelling [77].

#### 5.5.2. Beta‐Blockers

In suspected myocarditis, the absence of beta‐blocker therapy is linked to poorer outcomes [78, 79]. Bisoprolol or carvedilol improves prognosis in severe LVSD. In HFrEF, MERIT‐HF, COPERNICUS and CIBIS‐II showed mortality benefit in ischaemic and nonischaemic cardiomyopathy, excluding recent MI [80–82]; CAPRICORN demonstrated benefit with carvedilol after acute MI with LV dysfunction [83]. Withhold beta‐blockers in cardiogenic shock or significant hypotension, including early FM [78]. Preclinical data suggest agent‐specific effects: Carvedilol was more cardioprotective than metoprolol/propranolol in autoimmune myocarditis [84], whereas metoprolol increased inflammation, necrosis and death in acute murine Coxsackie B3 myocarditis [85].

#### 5.5.3. Aldosterone Antagonists

Eplerenone attenuated murine viral myocarditis by inhibiting mast cell‐derived proteinases and reduced fibrosis/remodelling [85]. Acute human myocarditis has been associated with high myocardial aldosterone synthase, suggesting aldosterone antagonism may reduce downstream HF/DCM [86].

#### 5.5.4. Sacubitril/Valsartan

Evidence supporting sacubitril/valsartan in myocarditis remains limited, despite demonstrated superiority over ACE inhibition in chronic LV systolic dysfunction (PARADIGM‐HF) [87]. In PIONEER‐HF (stable ADHF), sacubitril/valsartan achieved greater NT‐proBNP reduction than enalapril (−46.7% vs. −25.3%; HR 0.71, 95% CI 0.63–0.81; *p* < 0.001) with similar hypotension and hyperkalaemia. Reductions were seen within 1 week; troponin *T* fell and HF rehospitalisation was lower, although the trial was not powered for clinical endpoints [88]. TRANSITION showed feasible initiation predischarge (≥ 24 h after stabilisation) or Days 1–14 postdischarge; stabilised myocarditis was not excluded, and ∼50% reached target dose by 10 weeks [89].

#### 5.5.5. SGLT2 Inhibitors

Evidence supporting SGLT2 inhibitor use in myocarditis remains preclinical. Proposed benefits include reduced myocardial inflammatory cell infiltration, attenuation of profibrotic signalling, inhibition of inflammasome activation and preservation of LV systolic function [90–93]. These mechanistic properties suggest SGLT2i may represent a therapeutic adjunct in the management of acute myocarditis. Clinical efficacy in myocarditis remains unproven. Guideline‐directed SGLT2i therapy in myocarditis patients is currently limited to those meeting established HF treatment criteria.

#### 5.5.6. Diuretics

Intravenous furosemide remains first line for acute HF in the United Kingdom, with step‐down to oral furosemide or bumetanide (often better absorbed) once stable. Prior to discharge, euvolaemia should be maintained for 24–48 h after oral conversion. In rats with postautoimmune myocarditis, torasemide improved LV function and limited remodelling beyond renal effects [94].

### 5.6. Mechanical Circulatory Support and Heart Transplantation

**FIGURE 5:**
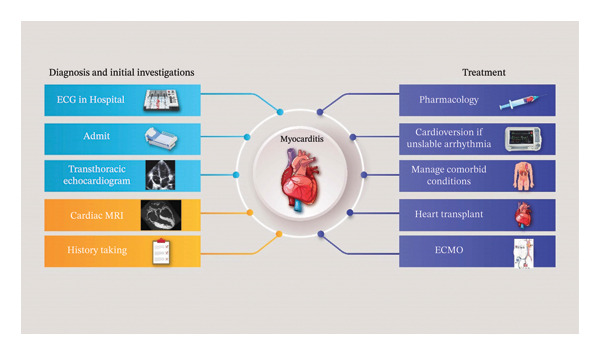
Summary of the workup and management for myocarditis. ECG: electrocardiogram. MRI: magnetic resonance imaging. ECMO: extracorporeal membrane oxygenation.

For patients with cardiogenic shock due to acute FM who deteriorate despite optimal medical treatment, mechanical circulatory support or extracorporeal membrane oxygenation may be required to bridge the patient to recovery or heart transplantation. Despite the severe initial presentation, these patients have a good prognosis and a high recovery rate of native ventricular function [22]. An overview of the diagnostic workup and treatment pathway for myocarditis is summarised in Figure 5.

### 5.7. Unloading Strategies

In FM complicated by cardiogenic shock, VA‐ECMO can provide rapid haemodynamic stabilisation, but it may increase LV afterload, predisposing to LV distension, pulmonary oedema, impaired myocardial recovery and intracardiac stasis/thrombosis [95–97]. LV unloading should be considered early when there is evidence of LV overload (e.g., worsening pulmonary oedema, low pulse pressure, absent or intermittent aortic valve opening, progressive LV dilatation or rising filling pressures on echocardiography/invasive monitoring) [95, 96, 98]. A pragmatic stepwise approach includes optimisation of medical strategies to promote LV ejection (careful preload/afterload management and inotropes/vasodilators where appropriate) alongside escalation to mechanical unloading when overload persists or complications develop [95–97]. Mechanical options include intra‐aortic balloon pump (IABP), percutaneous microaxial LV assist devices (e.g., Impella and ‘ECpella’) and atrial septostomy/transseptal left atrial (or surgical) venting; selection is individualised based on haemodynamics, ventricular loading, access/bleeding risk and local expertise [95, 98–100]. Observational data suggest associations between unloading and improved outcomes, although device‐related complications (notably bleeding/haemolysis/vascular injury) are increased, and randomised evidence has been mixed (e.g., EARLY‐UNLOAD showed no 30‐day mortality reduction with routine early transseptal unloading but faster resolution of pulmonary congestion) [98–101].

### 5.8. Physical Activity

Physical activity should be restricted during the acute phase of myocarditis until the disease has completely resolved. Athletes diagnosed with myocarditis should be excluded from exercise programmes for 3–6 months, depending on the severity and duration of the illness, the LV function at onset and the degree of inflammation on the CMR, according to a recent position statement issued by the Sport Cardiology Section of the European Association of Preventive Cardiology (EAPC) [102]. Athletes who have experienced myocarditis in the past should be periodically reassessed, especially in the first 2 years. After suffering myocarditis, athletes should be allowed to return to training and competition if their LV systolic function; blood test indicators of myocardial damage and exercise test results show no clinically significant abnormalities.

An athlete with clinically healed myocarditis may have chronic LV ecchymosis. However, the clinical relevance of this condition is unknown. Ventricular tachyarrhythmias may arise from a myocardial scar. As long as LV function is maintained and there are no frequent or complex repetitive forms of ventricular or supraventricular arrhythmias during maximal exercise and on 24‐h ECG monitoring (including training/competition sessions), it appears reasonable for these athletes to resume training and competitive sports. Nonetheless, asymptomatic athletes with LGE should continue receiving yearly clinical monitoring [102, 103].

Athletes with probable or definite myocarditis should be temporarily excluded from competitive and even amateur leisure activity regardless of age, gender, severity of symptoms and LV function [102–104]. Even after the resolution of acute inflammation, a risk of arrhythmias related to the resultant myocardial scar is present. The presence of LGE may indicate a heightened risk for arrhythmias. Currently, it remains unclear as to whether the resolution of myocarditis‐related LGE should be a prerequisite to permit a return to competitive sports.

### 5.9. Specific Forms of Myocarditis

#### 5.9.1. GCM

Patients with GCM who received immunosuppressive therapy may have a significantly improved survival compared to patients who do not receive immunosuppressive treatment. Among 22 patients treated with corticosteroids and cyclosporine, azathioprine or both therapies, the average transplant‐free survival was 12.3 months compared with an average of 3.0 months for the 30 patients who received no immunosuppressive therapy. In a prospective study of 11 subjects who received high doses of steroids and cyclosporine, while nine subjects also received 7–10 days of muromonab‐CD3, during 1 year of treatment, one subject died of respiratory complications, and two subjects received HT [105].

Despite the possibility of fatal disease recurrence, transplantation is the treatment of choice for most patients. Among 34 patients who underwent transplantation for this disease, recurrence was documented in nine after an average follow‐up of three years. Of the 34 patients who underwent heart transplantation, 9 (26%) had a giant‐cell infiltrate in the transplanted heart after an average follow‐up of three years, and one died of recurrent giant‐cell myocarditis [105].

#### 5.9.2. Eosinophilic Myocarditis

In general, the treatment of eosinophilic myocarditis usually consists of high‐dose steroids and the removal of the offending drug (in the case of HSM) or treatment of the underlying disorder.

#### 5.9.3. ICI‐Associated Myocarditis

ICI‐associated myocarditis is a medical emergency. The ICI should be withheld immediately, and patients should be admitted to a monitored setting with early cardio‐oncology/oncology involvement [106–108]. In confirmed moderate–severe cases, permanent discontinuation of the culprit ICI is generally recommended [107, 108]. Most contemporary guidance supports prompt high‐dose corticosteroids, commonly IV methylprednisolone 500–1000 mg daily for 3–5 days, followed by high‐dose oral prednisone with a slow taper guided by symptoms, ECG/rhythm stability, ventricular function and serial troponin [106, 108]. The American Society of Clinical Oncology (ASCO) additionally recommends starting prednisone/methylprednisolone 1–2 mg/kg/day, with rapid escalation (including pulse dosing) if there is inadequate early response or clinical deterioration [107]. Observational data suggest that earlier initiation (≤ 24 h) and higher initial steroid dosing are associated with fewer major adverse cardiac events, supporting a low threshold for pulse therapy in moderate–severe presentations [109, 110]. In fulminant, refractory or steroid‐dependent disease, escalation to additional immunosuppression may be required, commonly mycophenolate mofetil, calcineurin inhibitors, IVIG, plasmaphaeresis and/or antithymocyte globulin, although evidence is largely observational and should be individualised with multidisciplinary input [106, 108, 111]. Infliximab has been used in selected refractory cases but should be approached cautiously (or avoided) in patients with significant LV dysfunction given HF safety concerns and limited supportive data [106–108]. Mechanism‐directed rescue strategies have also been reported, including abatacept (CTLA‐4‐Ig) and alemtuzumab [112, 113].

### 5.10. Withdrawal of Pharmacological Treatment Once Cardiac Function Normalises

Evidence remains limited on whether pharmacological therapy can be stopped after cardiac function normalises. From the few studies based on retrospective case‐note reviews of populations with varying levels of recovery, continuation of medical HF treatment in patients with recovered DCM is unclear [114–116]. Often, patients with myocarditis are young, and their disease is short‐lasting. Many of them are reluctant to take lifelong medications following the resolution of HF due to side effects. In the case of young women wishing to conceive, drugs like ACE inhibitors can cause foetal abnormalities. Without robust studies, patients are often given conflicting medical guidance.

TRED‐HF [117] was a single‐centre, open‐label UK study of 51 patients who had prior DCM and a median LVEF of 25% at the time of diagnosis 4.9 years earlier and who subsequently recovered in response to therapy. Twenty‐five patients were randomised to a phased withdrawal of their HF drugs over 16 weeks or continuation of HF medications. The primary endpoint was relapse of DCM within 6 months of the start of the study, defined as either a drop in LVEF of more than 10% to a level below 50%, at least a doubling of NT‐pro‐BNP to greater than 400 ng/L, clinical evidence of HF or a greater than 10% increase in LV end diastolic volume as assessed by CMR. The results of the trial suggest that around four in 10 patients with recovered DCM will have a relapse within 6 months of starting a phased withdrawal of pharmacological treatment for HF. Data from this randomised trial suggest that treatment should not usually be withdrawn in patients with recovered DCM [117]. Further research might identify subgroups of patients, such as those with myocarditis, in whom pharmacological treatment for HF can be safely withdrawn.

## 6. The COVID‐19–Related Myocarditis

The COVID‐19 pandemic has drawn renewed attention to myocarditis, both as a complication of SARS‐CoV‐2 infection and, rarely, following vaccination. Myocardial injury in COVID‐19 may occur through several mechanisms, including direct viral invasion of cardiomyocytes via ACE2 receptors, immune‐mediated injury, microvascular dysfunction and systemic inflammatory activation (‘cytokine storm’) [118]. Clinical presentations range from mild chest pain with troponin elevation to FM with cardiogenic shock.

Early case series reported myocardial inflammation in a subset of hospitalised patients with COVID‐19, frequently with elevated cardiac biomarkers and, where performed, abnormal CMR findings. FM has been documented, with some patients responding to high‐dose corticosteroids and/or intravenous immunoglobulin alongside supportive therapy. However, histopathological confirmation was rare, and distinguishing direct viral myocarditis from secondary myocardial injury remains challenging.

Myocarditis has also been reported following mRNA‐based COVID‐19 vaccination, with the highest absolute risk in males aged 12–29 years, typically occurring within one week after the second dose [119]. Most cases have a benign course with symptom resolution and preserved cardiac function on follow‐up, though CMR may show LGE in some patients [120]. Large population‐based studies confirm that SARS‐CoV‐2 infection carries a higher myocarditis risk than vaccination [121].

Management of COVID‐19–related myocarditis follows standard principles for acute myocarditis, with supportive HF therapy, arrhythmia management and restriction of physical activity during the acute phase. Immunomodulatory therapies may be considered in fulminant cases with suspected immune‐mediated injury, but robust trial data are lacking.

Despite these occurrences, the current body of evidence strongly supports that the benefits of COVID‐19 vaccination significantly outweigh the associated risks. Nonetheless, ongoing active surveillance remains essential to ensure vaccine safety [122–125].

## 7. Recurrence and Genetics

A subset of patients may experience recurrent myocarditis, which can result from persistent viral infection, autoimmune mechanisms or incomplete resolution of inflammation [2, 8, 126]. Recurrence is more frequently observed in individuals with underlying autoimmune diseases [126], those with genetic predispositions such as variants in desmosomal or sarcomeric genes [127] and younger patients under the age of 40 who have a history of myocarditis and preserved cardiac function [8].

Advancements in next‐generation sequencing and familial screening have uncovered a shared genetic architecture between myocarditis and inherited cardiomyopathies, including titin (TTN)–truncating variants and desmoplakin (DSP) mutations. These findings support the consideration of genetic evaluation in patients with relapsing or familial forms of the disease [127].

## 8. Future Research

Several areas require further research in myocarditis. More studies are needed to improve our understanding of the potential role genetic susceptibility can play in the development of the condition [128, 129]. Several studies have already indicated a correlation between myocarditis and certain genetic backgrounds. For example, pathogenic genetic variants linked to dilated or arrhythmogenic cardiomyopathy, such as the DSP gene and the TTN gene, were found in 8%–16% of myocarditis patients [128–135]. This has also been supported by certain clinical traits such as neurosensory disorders, skeletal muscle involvement, creatine kinase elevation and persistent left bundle block or AV block [128]. The latest research also highlighted the prognostic role of pathogenic mutations in creating vulnerable myocardium prone to inflammation [128, 136]. This can lead to the development of recurrent pericarditis and a higher incidence of more serious complications like ventricular arrhythmia and HF [129, 134]. Further, large multicentre studies are needed to better identify these genetic markers and high‐risk patients requiring genetic testing. This can improve the diagnosis, risk stratification and management of myocarditis patients, paving the way for a more individualised and risk‐tailored approach [128, 129].

Another promising avenue relates to the role that gut microbiota may play a role in the development of myocarditis [137]. In recent years, human gut microbiota has gained significant attention, with studies showing its contribution to the occurrence and development of cardiovascular diseases, including hypertension, atherosclerosis, HF, arrhythmias and cardiomyopathy [137–145]. Recent studies have also revealed that gut microbiota can be associated with myocarditis [137, 146–150]. The mechanisms by which microbiota promote myocarditis are complex and involve immune system dysregulation, inflammation and endothelial dysfunction. Further research exploring the gut microbiota–immune system–myocarditis axis can pave the way for identifying new viable therapeutic targets. Modulating gut microbiota through probiotics, prebiotics, symbiotics, antibiotics or faecal transplantation represents promising future strategies for myocarditis treatment [137].

Expanding the roles anti‐inflammatory therapy can play in improving prognosis in myocarditis is another active area of research. In acute myocarditis, the loss of immune tolerance generally initiates the aberrant immune response directed against myocytes [151]. This is primarily supported by T‐cell activation and B cells’ autoantibody production, along with NLRP3 inflammasome activation and IL‐1 release [151, 152]. The MYTHS trial (Myocarditis Therapy with Steroids) aims to define the role of intravenous methylprednisolone in the initial treatment of complicated forms of acute myocarditis. The safety and efficacy of pulsed intravenous methylprednisolone versus placebo on top of standard therapy and maximal supportive care will be tested [151, 153]. The Phase 3 ARGO trial (Colchicine vs Placebo in Acute Myocarditis Patients) tests the effects of 6 months of colchicine treatment on CMR parameters in uncomplicated acute pericarditis [151]. The CMP‐MYTHiC trial (Cardiomyopathy with Myocarditis Therapy with Colchicine) will assess the efficacy of colchicine in reducing complications in chronic myocarditis patients [154].

There remain gaps in the evidence base regarding the period of exercise restriction for athletes postmyocarditis [155]. The current ESC and AHA/ACC guidelines recommend exercise restriction for 3–6 months, following an assessment including Holter monitoring, echocardiogram, exercise ECG and CMR [103, 155, 156]. However, these recommendations are mainly based on expert opinion, as shown in the recommendation level, Class IIa Level C [155, 156]. The clinical significance of LGE persistence in those who remain asymptomatic post the index event remains an existing knowledge gap [103, 155]. Therefore, prospective longitudinal studies tracking recovery outcomes and trajectories in athletes following acute myocarditis are warranted to support the development of robust evidence‐based protocols. This is particularly important for elite athletes whose careers depend on physical performance [155].

## 9. Role of Artificial Intelligence (AI)

AI and machine learning (ML) are increasingly used in cardiology and may improve myocarditis diagnosis and prognostication, particularly through CMR image analysis. Recent studies have applied deep learning and radiomics techniques to detect subtle myocardial texture changes and to differentiate myocarditis from myocardial infarction or takotsubo cardiomyopathy [157].

For example, Cavallo et al. demonstrated that radiomics features from short‐tau inversion recovery (STIR) images could predict LGH presence in acute myocarditis with an accuracy of 84% in a single‐centre cohort [158]. Similarly, Di Noto et al. reported that radiomics analysis outperformed visual inspection for differentiating myocarditis from infarction on LGE imaging [158].

AI models have also been developed using parametric mapping data (native T1, T2 and ECV) as inputs. Shyam‐Sundar et al. applied convolutional neural networks (CNNs) to T1 and T2 maps in suspected myocarditis, reporting improved sensitivity compared with the 2009 LLC in a retrospective dataset [159]. However, these findings require validation in multicentre prospective studies, as most published work is based on small, single‐centre cohorts.

Beyond diagnosis, preliminary work has explored AI‐driven risk stratification, integrating imaging with clinical and biomarker data to predict adverse events such as ventricular arrhythmia or persistent LV dysfunction [160]. These approaches remain experimental, and there is a recognised need for transparent model reporting and external validation. Recent systematic reviews have emphasised the importance of standardised reporting frameworks such as TRIPOD‐AI for prediction models and CONSORT‐AI for interventional trials [160].

Importantly, the current AI evidence base in myocarditis is limited by small retrospective datasets, frequent single‐centre design, selection and spectrum bias, heterogeneity in CMR acquisition protocols and diagnostic definitions and limited biopsy‐confirmed ground truth. External validation across different scanners, institutions, populations and clinical pathways remains scarce [159, 160].

Current challenges also include dataset heterogeneity and the ‘black box’ nature of some deep learning models, which may reduce clinician trust. To address these, explainable AI techniques and multimodal model training are emerging as priorities for future research [161]. Therefore, although AI‐based approaches may support future diagnostic and prognostic workflows, they should currently be considered investigational rather than ready for routine clinical implementation. While AI offers promise in streamlining myocarditis workup and potentially identifying prognostic phenotypes, its integration into clinical pathways should await robust prospective validation in diverse populations.

## 10. Conclusion

Myocarditis remains a complex clinical challenge due to its heterogeneous presentation, evolving diagnostic criteria and diverse management strategies. While the traditional reliance on invasive diagnostic methods has declined, advanced noninvasive imaging techniques, especially CMR with novel parametric mapping, have enhanced clinical diagnosis and prognostication. Treatment remains centred on supportive HF and arrhythmia care, with selected use of immunosuppression, antiviral therapy or mechanical circulatory support according to myocarditis subtype and severity. The COVID‐19 pandemic has further complicated the clinical landscape, highlighting myocarditis as both a direct complication of SARS‐CoV‐2 infection and a rare but important side effect of vaccination. Ongoing research is essential to refine diagnostic protocols, optimise management strategies and mitigate risks associated with myocarditis in COVID‐19.

## Author Contributions

Kevin Mohee, Sanjay S. Bhandari and Ibrahim Antoun: conceptualisation, methodology, validation and writing original manuscript. Kevin Mohee, Ibrahim Antoun, Sanjay S. Bhandari, Robert Ambrogetti, Mahmoud Eldesouky, André Ng and Riyaz Somani: writing–review and editing.

## Funding

The authors have nothing to report.

## Conflicts of Interest

The authors declare no conflicts of interest.

## Data Availability

No original research has been carried out, hence no data were generated.
